# Effect of Copper and Zinc Ions on Biochemical and Molecular Characteristics of Calcium Oxalate Renal Stones: a Controlled Clinical Study

**DOI:** 10.1007/s12011-023-03686-0

**Published:** 2023-05-16

**Authors:** Shaimaa A. Y. Taha, Ahmed A. Shokeir, Wael I. Mortada, Amira Awadalla, Lamiaa A. A. Barakat

**Affiliations:** 1 Center of Excellence for Genome and Cancer Research, Urology and Nephrology Center, Mansoura University, Mansoura, 35516 Egypt; 2 Clinical Chemistry Laboratory, Urology and Nephrology Center, Mansoura University, Mansoura, 35516 Egypt; 3 Department of Biochemistry, Faculty of Science, Port Said University, Port Said, Egypt

**Keywords:** Calcium oxalate stones, Copper, Zinc, Oxidative stress, ERK, P38, JNK

## Abstract

Contradictory results are existed in the literature regarding the impact of trace elements on the pathogenesis of calcium oxalate (CaOx) stone patients. Therefore, the aim of our study was to investigate the effect of Cu and Zn on biochemical and molecular characteristics of CaOx stones. Plasma and urine concentrations of Cu and Zn in 30 CaOx stones patients and 20 controls were determined by flame atomic absorption spectrometry (FAAS). Urinary levels of citric acid and oxalate were measured by commercial spectrophotometric kits. Blood levels of glutathione reduced (GSH) and catalase (CAT) were determined as markers of antioxidant activity, while blood malondialdehyde (MDA) and urine level of nitric oxide (NO) were used to assess oxidative stress. Gene expression of MAPk pathway (ERK, P38, and JNK) were estimated. The plasma and urine levels of Cu were significantly increased in the patient group compared to those of controls, while the levels of Zn were decreased. Excessive urinary excretion of citric acid and oxalate were found among CaOx stone patients. The GSH and CAT concentration were significantly reduced in CaOx stones patients compared to healthy group. The plasma MDA and urine NO concentration were significantly increased in CaOx stones patients compared to control group. The expressions of the studied genes were significantly increased in CaOx stones patients. These findings suggest that alteration in Cu and Zn might contribute to pathogenesis of CaOx patients through oxidative stress and MAPK pathway genes (ERK, P38 and JNK).

## Introduction

Kidney stone disease affects about 15–20% of the population and accounts for a major portion of global health-care costs [1]. If not treated appropriately, it might permanently damage the kidneys. The disease is more common in the Afro-Asian region [2].

One of the most prevalent components of kidney stones, calcium oxalate (CaOx), is implicated in the production of 80% of renal stones [3]. Damage to renal tubular epithelial cells and changes in crystal adhesion in renal tubular epithelial cells have been identified as essential factors in the production of CaOx stones [4, 5]. Environmental, genetic, and oxidative stress variables are thought to have a role in the disease's development. The fundamental process causing the shift in crystal adhesion in renal tubular epithelial cells, however, remains unknown.

Several studies have investigated the role of trace elements in the development of stone disease. However, the results of the studies on trace elements (such as copper (Cu) and Zinc (Zn)) are not consistent [6]. However, the significance of trace metals in the pathophysiology of kidney stones is unknown. According to certain research, dietary Zn consumption may be related with an increased risk of kidney stones, but manganese(Mn) intake may be associated with a lower risk of kidney stones [7]. Another study concluded that intake of Zn and iron was not related to a higher incidence of kidney stones. However, Cu intake may be correlated to an increased risk in some people [8]. Chi and colleagues explored that mineral concretions formed after inhibiting xanthine dehydrogenase were rich in Zn; additionally, inhibition of Zn transporter genes in the same model suppressed stone formation, implying that Zn may play a critical role in driving the process of heterogeneous nucleation [9]. According to the National Health and Nutrition Examination Survey, participants with a self-reported history of kidney stones had greater dietary Zn consumption [10]. A small case–control study, on the other hand, found reduced Zn intakes among individuals with stones [11]. Some investigations suggest that some major and trace elements have a role in the genesis of stone crystallization, either as a nucleus or nidus for the development of the stone or merely as an impurity of the stone structure [12].

P38 Mitogen-activated protein kinase (P38 MAPK) played a role in the development of atherosclerosis by influencing collagen expression directly [13]. Paleerath et al. found that the p38 MAPK signaling pathway was involved in the breakdown of tight junctions in epithelial cells, and that the expression of associated p38 MAPK signaling pathway proteins was up-regulated during CaOx stone formation [14]. The oxalate dramatically increased p38 MAPK activity considerably, increased c-Jun NH2-terminal kinase (JNK) pathway activity and phosphorylation slightly, and had no effect on extracellular signal-regulated kinase (ERK) pathway activity and phosphorylation [14].

Furthermore, CaOx crystals have been shown to enhance lipid peroxidation and free oxygen radicals, which mediate crystal development and adhesion to epithelial tubules, as well as renal tubule cell injury [15, 16]. Lipid peroxidation is oxidative tissue damage caused by oxygen free radicals and their biological products, such as superoxide, hydroxyl radicals, and hydrogen peroxide.

Although the fact that various studies have highlighted the potential roles of the above parameters and their significance in the development of stone disease, no clinical studies have linked changes in trace element levels (e.g., Cu and Zn) to the previously listed factors. The objective of the present study is to investigate the effect of Cu and Zn ions on biochemical and molecular characteristics of CaOx renal stones through a controlled clinical study.

## Subjects and Methods

### Subjects

This is a controlled clinical study that was carried out at the Mansoura Urology and Nephrology Center at Mansoura University after approval of the local ethical committee (MS.21.08.1605). The study included 30 consecutive patients with CaOx stones confirmed by Fourier transform infrared spectroscopy (FT-IR) and matched 20 healthy controls free of stone disease.

### Collection of Urine and Blood Samples

All individuals were asked to provide 24-h urine samples. The patient was instructed to discard the first voided morning sample and start to collect the 24 h urine including the first voided urine in the morning of the following day. The urine samples were centrifuged at 3000 rpm for 10 min and analyzed for citrate, oxalate, nitric oxide (NO), Cu, and Zn levels. Blood samples were also collected from all individuals on tubes containing K_2_EDTA as an anticoagulant. A part of samples was used to detect the gene expression levels of the ERK, P38, and JNK genes. The other part was centrifuged at 4000 rpm for 10 min and the plasma was analyzed for Cu, Zn, reduced glutathione (GSH) concentration, catalase (CAT) activity, and malondialdehyde (MDA) concentration.

### Methods

#### Determination of Cu and Zn Levels

Plasma and urine samples were digested as follows: 1.0 mL of plasma or 2.0 mL of urine samples, 3 mL HNO_3_, and 1 mL H_2_O_2_ were mixed in the digestion level and allowed to stand at room temperature for 15 min. The tubes were heated in a microwave oven (Speed wave four, Berghof Products, Germany) using a one-stage digestion program as follows: 1600 W (100%); 15-min ramp; 200 °C temperature; 15-min hold; and 15 min cooling [17]. After cooling, the solutions were diluted to 10 mL using deionized water then analyzed for Cu and Zn by using a Buck Scientific atomic absorption spectrometer (model 210 VCP, East Norwalk, CT, USA) equipped with air/acetylene flame and hollow cathode lamps for Cu and Zn at wavelengths of 324.8 and 213.9 nm, respectively and spectral bandwidth of 0.7 nm. Analysis of spiked samples was used to test the procedure accuracy, and the recovery rate was in the range of 97.5–99.0%. The precision did not exceed 3.0 percent (in terms of relative standard deviation).

#### Determination of Citric Acid and Oxalate in Urine

A 24-h urine sample was immediately centrifuged at 3000 rpm for 10 min at 4 °C. The supernatant was divided into aliquots and frozen at 80 °C in 1.5-mL tubes. Citric acid and oxalate were measured using the manual kits purchased from (Biochemical Enterprise, italy) [18].

#### Determination of Antioxidants (GSH, CAT) and Oxidative Stress (MDA, NO) Levels

The reduced glutathione (GSH) concentration, catalase (CAT) activity, and malondialdehyde (MDA) amount were detected in plasma samples by Bio-Diagnostic commercial kits (Giza, Egypt) according manufacturer [19]. Similarly, in urine samples nitric oxide (NO) concentration was measured [20]. A 7300 Genway spectrophotometer was used for all spectrophotometric measurements (Cole-Parmer Ltd., Staffordshire, UK).

#### Gene Expression Assay for ERK, P38, and JNK genes

QIAamp RNA Blood Mini Kit (supplied by QIAGEN Cat.NO.52304., USA) was used to extract the RNA from blood samples. The concentration and purity of RNA samples were determined by using a Thermo Scientific NanoDropspectrophotometer model 2000c (NanoDrop Technologies, Wilmington, idiumbromide(. The High Capacity cDNA reverse transcription Kit was used to convert RNA samples to complementary DNA (cDNA) (Thermo Fisher Scientific, Waltham, MA, USA(. cDNA samples were preserved at − 80 °C. SYPER Green PCR Master Mix was used for quantitative RT-PCR (Thermo Fisher Scientific, Waltham, MA, USA.(The mRNA expression levels of ERK, P38, JNK, and also GAPDH as a housekeeping gene (internal control) were measured using Step one plus real-time PCR (Applied Biosystems). The tests were carried out in triplicate. The primer sequences for the genes investigated are included in Table 1. The following programmer is used to adjust the PCR cycle parameters: pre-denaturation at 95 °C for 10 min, 40 cycles in denaturation at 95 °C for 15 s, annealing at 60 °C for 1 min, and finally extension at 72 °C for 1 min.Using this equation **RQ = 2**^**−ΔΔCT**^, to calculate the relative quantification [17].

**Table 1:** List of primer sequence

Gene	Sequence	Product length(bp)	Accession no
P38	F:5-GCATAATGGCCGAGCTGTTG -3	130	NM_001315.3
	R:5-TCATGGCTTGGCATCCTGTT-3		
JNK	F:5-TTGGAACACCATGTCCTGAA-3	183	NM_001278547.2
	R:5-ATGTACGGGTGTTGGAGAGC-3		
ERK	F: 5- ATCGCCGAAGCACCATTCAA-3	194	NM_002745.5
	R: 5-AGGACCAGGGGTCAAGAACT-3		

#### Statistical Investigation

Continuous variables were presented as mean ± SD and categorical variables as frequency and percentage. Student’s *t*-test and Chi square test were used as appropriate. The correlation between the continuous variables of both groups was calculated by Pearson coefficient correlation analysis with determination of r value. Results interpreted as strong correlation with *r* (0.7–1), moderate (0.3–0.7), weak (0.1–0.3), and no correlation (< 0.1). A software SPSS version 20 was used for statistical analysis of data (MAS Medical and Scientific Eq. Co, IL, USA), while Excel 2010 (Microsoft Office) was used for diagram production.

## Results

Thirty patients with CaOx stones (15 males and 15 females) with a mean age of 51.4 ± 13.57 years were considered for the study. The controls consisted of 20 healthy individuals (10 males and 10 females) with a mean age of 53.2 ± 7.14 years. Both groups were matched in terms of age, gender, BMI, and diabetic status (Table 2).

**Table 2:** Patients characteristics

Item	CaOx patients	Control group	*p* value
Number	30	20	
Age (Years) mean ± SD	51.4 ± 13.57	53.2 ± 7.14	0.596
Gender (n, %)			
Male Female	15(50) 15(50)	10(50) 10(50)	0.613
BMI (kg/m2) mean ± SD	33.6 ± 7.94	33.5 ± 6.24	0.914
Diabetes, (n, %)	6(20)	6(30)	0.506
Serum creatinine (mg dL^−1^) mean ± SD	1.08 ± 0.46	0.93 ± 0.2	0.262
Uric acid (mg dL^−1^) mean ± SD	6.29 ± 1.8	5.6 ± 1.14	0.145

### Copper and Zinc levels in the Plasma and Urine

Table 3 shows plasma and urine Cu and Zn levels and Cu/Zn ratio in CaOx patients and control groups. CaOx patients have significantly higher plasma and urine Cu levels as well as a higher Cu/Zn ratio (*p* < 0.001) as compared to the control. Alternatively, levels of Zn in the plasma and urine in CaOx patients are considerably less than in the control group (*p* < 0.001).

**Table 3:** Comparison between CaOx stone patients and control groups in the study parameters

Marker	Ca oxalate patients	Control group	*p* value
Cu and Zn in blood and urine, mean ± SD			
Cu(µg/L) in plasma	114.2 ± 26.9	71.5 ± 16.9	0.001
Cu(µg/L) in urine	131.7 ± 34.1	73.6 ± 15.8	0.001
Zn(µg/L) in plasma	7.9 ± 2.8	13.6 ± 3.8	0.001
Zn(µg/L) in urine	5.8 ± 1.4	14.4 ± 3.8	0.001
Cu/Zn ratio in urine	23.8 ± 8.1	5.4 ± 1.5	0.001
Cu/Zn ratio in plasma	16.3 ± 7.5	5.6 ± 1.8	0.001
Citric acid and oxalate in urine, mean ± SD			
Citric acid (mg/24 h)	513.1 ± 114.3	215.4 ± 50.1	0.001
Oxalate(mg/24 h)	19.2 ± 4.3	13.2 ± 2.6	0.001
Antioxidant and oxidative stress markers in the blood, mean ± SD			
GSH(mg/dl)	6.1 ± 2.2	14.7 ± 3.6	0.001
MDA(nmol/ml)	8.5 ± 2.1	2.7 ± 0.9	0.001
NO (µmol/L)	9.5 ± 3.4	2.5 ± 0.81	0.001
CAT (u/ml)	0.6 ± 0.13	0.8 ± 0.13	0.001
Gene expression by real time PCR (RT-PCR) in the blood, mean ± SD			
ERK	4.5 ± 1.3	1.03 ± 0.01	0.001
P38	1.89 ± 0.58	0.94 ± 0.12	0.001
JNK	2.81 ± 0.63	1.01 ± 0.06	0.001

**Fig. 1:**
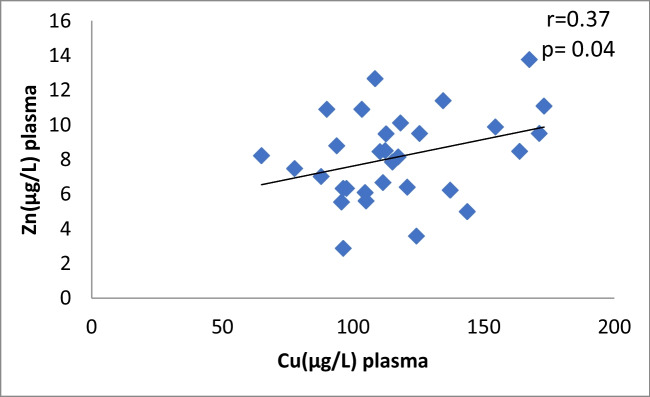
Scatter plot of plasma Cu and Zn in Caox patients

In patients with CaOx stones, there is a significant moderately positive correlation between Cu and Zn in the plasma (*r* = 0.37, *p* = 0.04) shown in Fig. 1, urine Cu and urine Zn showed no correlation (*r* =  − 0.04, *p* = 0.82). Moreover, there is no correlation between plasma Cu and urine Cu (*r* = 0.09, *p* = 0.62) and no correlation between plasma Zn and urine Zn (*r* = 0.06, *p* = 0.73) (Table 4). The control group showed no significant correlation between Cu and Zn neither in plasma nor in urine (Table 4).

**Table 4:** Pearson correlation Coefficients between different parameters in CaOx patients and control group

The correlation	CaOx patients			Control group		
	value of r	value of *p*	Interpretation	value of r	value of *p*	Interpretation
Cu *vs* Zn in plasma	0.37	0.04	Moderate positive	0.279	0.234	No correlation
Cu *vs* Zn in urine	-0.04	0.82	No correlation	-0.16	0.5	No correlation
Cu in plasma *vs* urine	0.09	0.62	No correlation	-0.285	0.224	No correlation
Zn in plasma *vs* urine	0.06	0.73	No correlation	-0.305	0.191	No correlation
Cu in plasma *vs* CAT	0.44	0.01	Moderate positive	0.149	0.532	No correlation
Zn in plasma *vs* CAT	0.37	0.04	Moderate positive	0.092	0.701	No correlation
Cu in plasma *vs* ERK	0.38	0.03	Moderate positive	0.257	0.274	No correlation
Cu in plasma *vs* P38	-0.38	0.02	Moderate negative	0.207	0.382	No correlation
Cu in plasma *vs* JNK	0.37	0.04	Moderate positive	-0.145	0.542	No correlation
plasma Zn *vs* ERK	0.13	0.47	No correlation	0.264	0.261	No correlation
plasma Zn *vs* P38	-0.26	0.16	No correlation	-0.114	0.633	No correlation
plasma Zn *vs* JNK	0.1	0.77	No correlation	0.322	0.166	No correlation

^*^Person test

### Levels of Citric Acid and Oxalate in Urine

In comparison to the control group, urinary citric acid and oxalate concentrations were significantly higher in CaOx patients (*p* < 0.001) as presented shown in Table 3.

### Antioxidants (GSH, CAT) and Oxidative Stress (MDA, NO) Levels

Table 3 shows that the GSH and CAT concentrations were significantly lower in patients with CaOx compared to the controls, while MDA and NO concentrations were higher in CaOx patients compared to controls (*p* < 0.001).

**Fig. 2:**
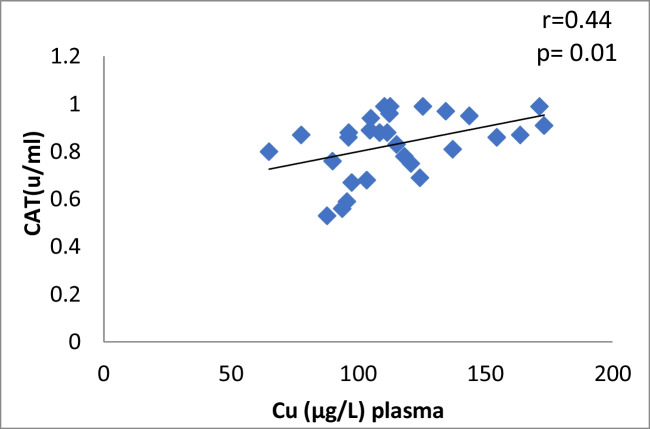
Plasma Cu against CAT in Ca stone patients

**Fig. 3:**
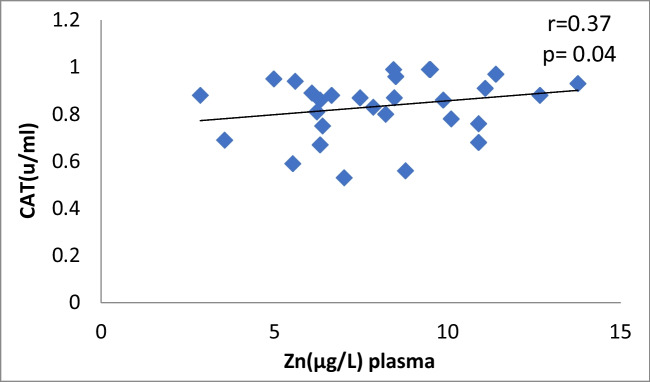
Scatter plot of plasma Zn against CAT in Ca oxalate patients

The correlation between plasma Cu and CAT levels in Ca oxalate patients showed a significant moderate positive (r = 0.44, *p* = 0.01) (Fig. 2). The data manifested a significant moderate positive correlation between plasma Zn and CAT activity in CaOx patients (*r* = 0.37, *p* = 0.04) (Table 4, Fig. 3). There is no correlation between antioxidants and oxidative stress markers and the levels of Cu and Zn in control subjects (Table 4).

### Genetic Results of ERK, P38, and JNK by Real-Time PCR

**Fig. 4:**
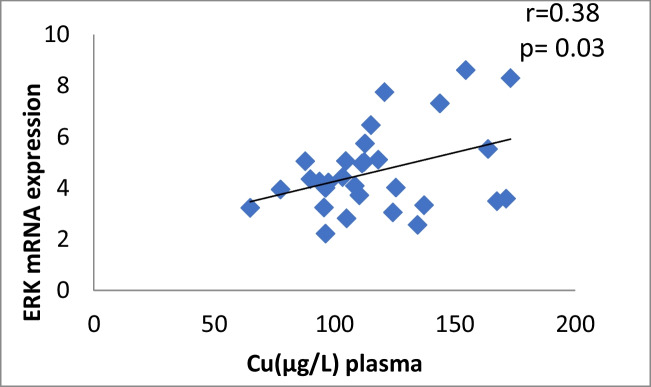
Scatter plot of plasma Cu against ERK mRNA expression inCaOx patients

**Fig. 5:**
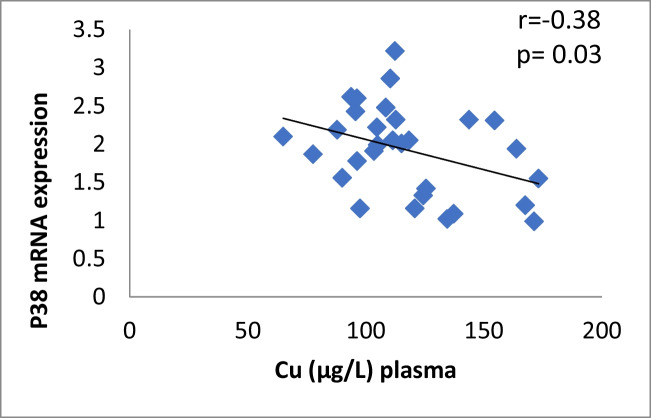
Scatter plot of plasma Cu against P38 mRNA expression in Ca stone patients

**Fig. 6:**
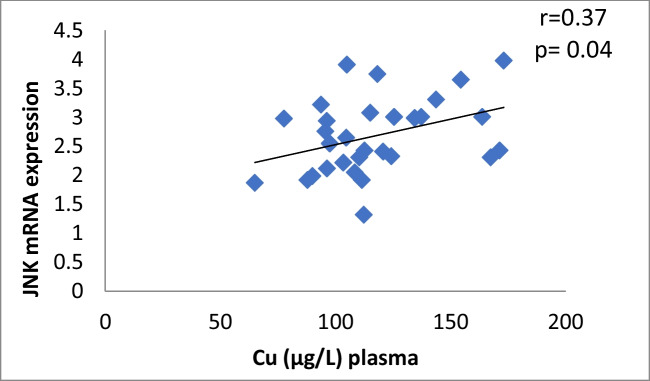
Scatter plot of plasma Cu against JNK mRNA expression in Ca oxalate patients

**Fig. 7:**
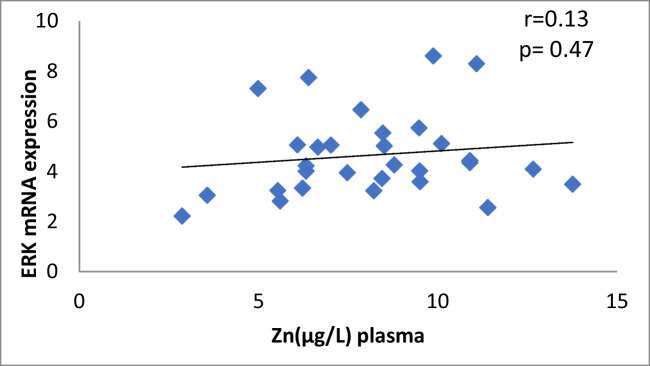
Scatter plot of plasma Zn against ERK mRNA expression in Ca oxalate patients

**Fig. 8:**
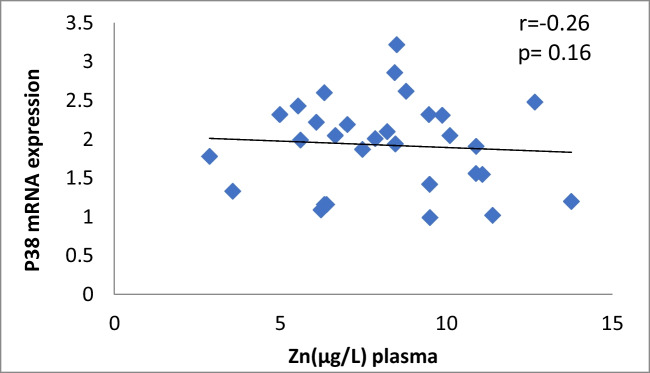
Scatter plot of plasma Zn against P38 mRNA expression in Ca oxalate patients

**Fig. 9:**
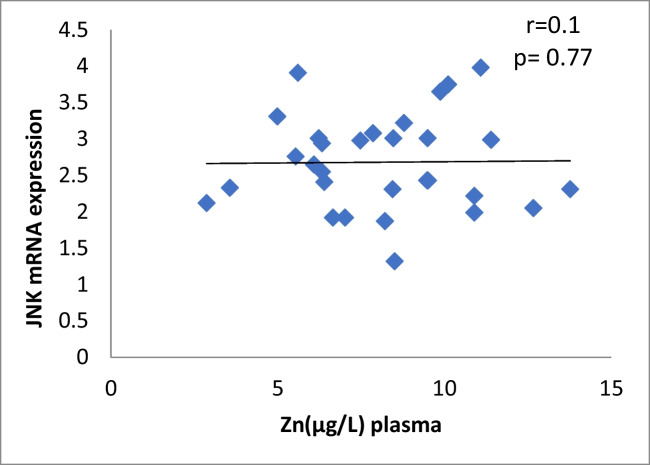
Scatter plot of plasma Zn against JNK mRNA expression in Ca oxalate patients

**Fig. 10:**
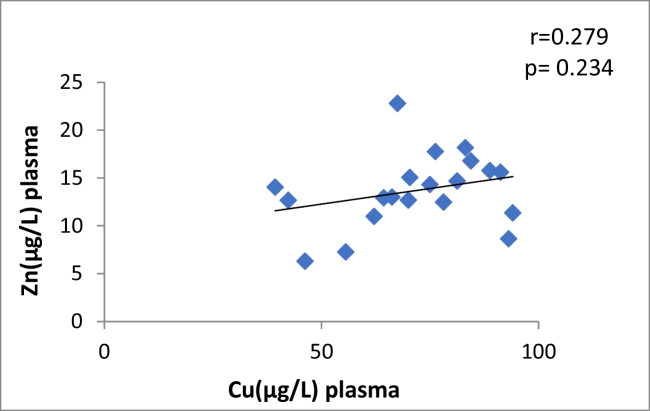
Scatter plot of plasma Cu and Zn in controls

**Fig. 11:**
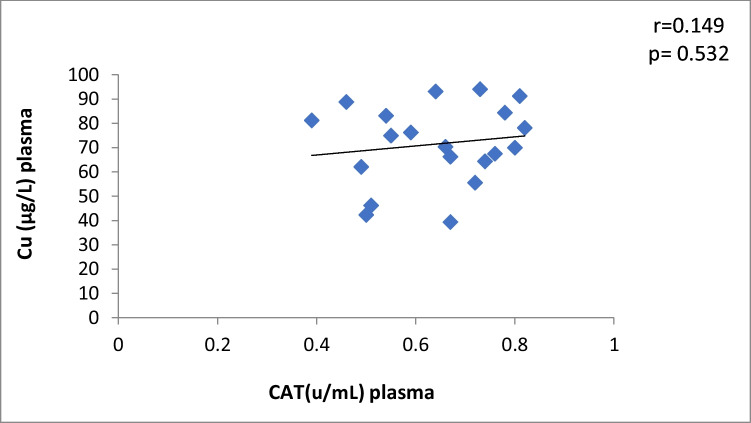
Scatter plot of plasma Cu and CAT in controls

**Fig. 12:**
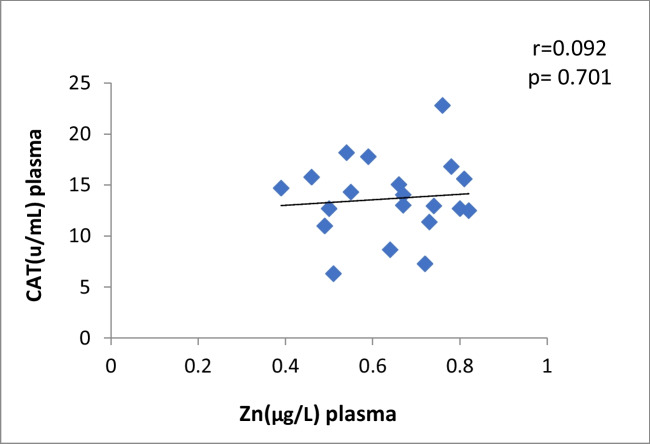
Scatter plot of plasma Zn and CAT in controls

**Fig. 13:**
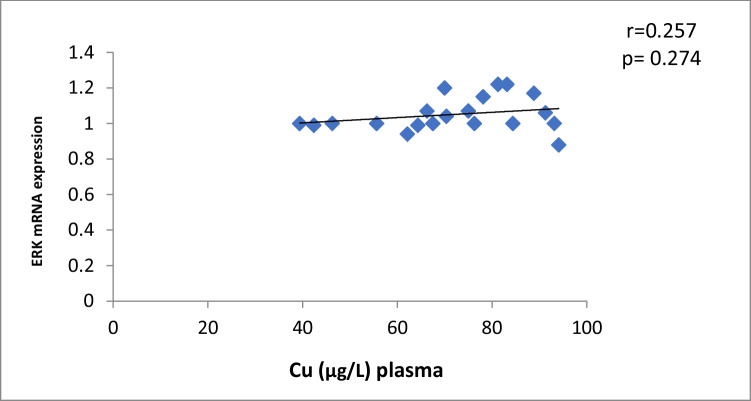
Scatter plot of plasma Cu against ERK mRNA expression in controls

**Fig. 14:**
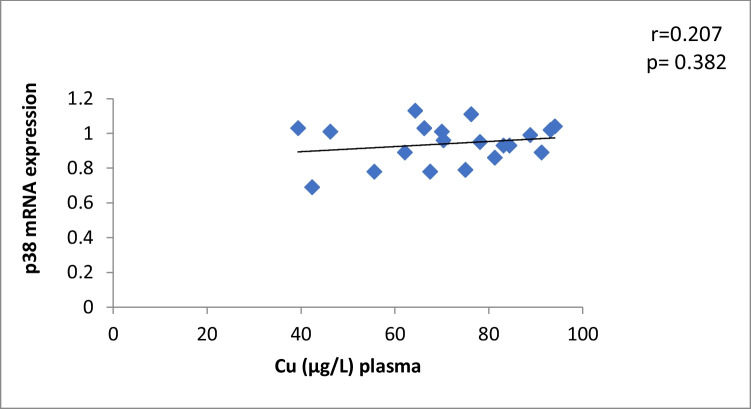
Scatter plot of plasma Cu against p38 mRNA expression in controls

**Fig. 15:**
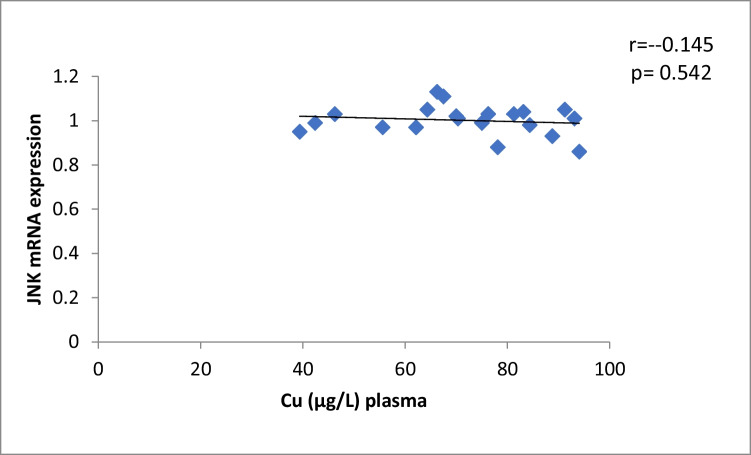
Scatter plot of plasma Cu against JNK mRNA expression in controls

**Fig. 16:**
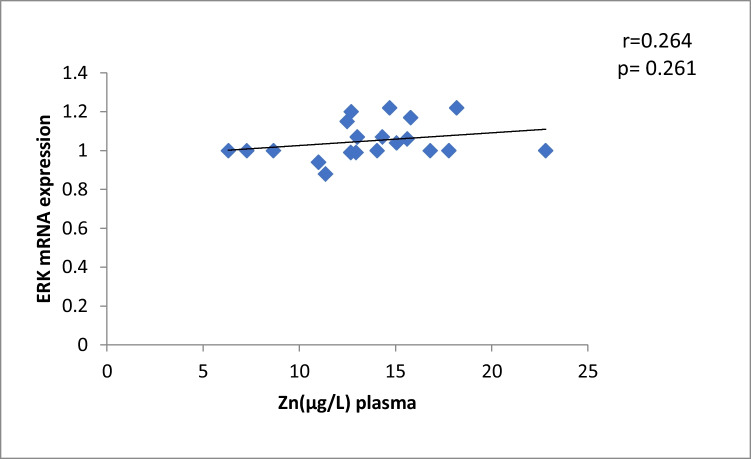
Scatter plot of plasma Zn against ERK mRNA expression in controls

**Fig. 17:**
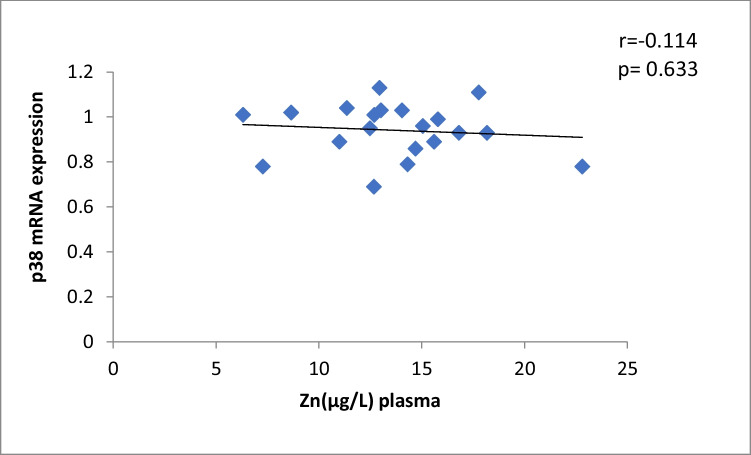
Scatter plot of plasma Zn against p38 mRNA expression in controls

**Fig. 18:**
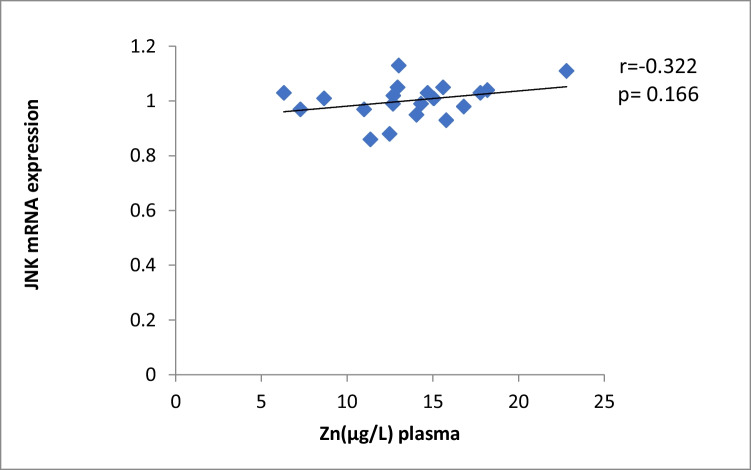
Scatter plot of plasma Zn against JNK mRNA expression in controls

The results of our study (Table 3) indicated that the expression of ERK, P38 and JNK were considerably higher than in control group (*p* < 0.001). Cu levels in plasma revealed a moderately positive correlation with ERK and JNK mRNA expression (*r* = 0.38, 0.37; *p* = 0.03, 0.04, respectively). On the other hand, a moderately negative correlation was observed for P38 expression (*r* = -0.38, *p* = 0.02) (Table 4, Figs. 4, 5, 6). Zn levels in plasma showed no correlation with ERK, JNK and P38 mRNA expression (*r* = 0.13, 0.1; *p* = 0.47, 0.77; *r* =  − 0.26; *p* = 0.16, respectively) (Table 4, Figs. 7, 8, 9, 10, 11, 12). There is no correlation between mRNAs expression levels and the levels of Cu and Zn in control subjects (Table 4, Figs. 13, 14, 15, 16, 17, 18).

## Discussion

Controversial results are existing in the literature regarding the role of the trace elements on the CaOx stone pathogenesis. In the present study, we tried to shed the light on the effect of Cu and Zn on biochemical and molecular characteristics of CaOx stones in a controlled clinical study.

The current study showed that the urine citric acid content in CaOx patients was higher than in controls. This result is disagreeing with Huang et al. [21], who found that the urinary citric acid concentration in CaOx patients was significantly lower than in the healthy group. Our results regarding the low level of citrate in the study group is contradictory to the standard information that citrate is an inhibitor of crystallization. Therefore, it is supposed to show increase instead of decrease in the study group compared to controls. Nevertheless, this contradictory finding may be explained by the fact that the level of citric acid is influenced by the diet, medication, and lifestyle variations [22]. These co-variables together with the small sample size may explain this contradictory result. On the other hand, the level of urine oxalate was considerably higher in CaOx patients compared to the control group. This result is consisting with the results obtained by other researchers [21]. The high level of 24-h urinary oxalate causes an increase in the supersaturation of the CaOx solid phase, which leads to the production of CaOx stones [23].

Zn is considered as the second most common trace element in the human body. It has been described as an inhibitor of urinary stone development [6]. The Zn ions could chelate with oxalate ions, lowering oxalate activity and causing a decrease in nucleation rates [24]. In the present study, Zn concentrations in urine and plasma were shown to be considerably lower in CaOx stone patients as compared to control group. Our results are in line with Atakan et al. [6] who investigated the urine and plasma levels of Zn in CaOx stones patients and healthy individuals. They found that zinc was significantly lower in stone patients compared to healthy controls.

Cu enhances the crystallization of stone and affect calcium oxalate growth at very low concentrations, through forming insoluble salts with oxalate ions [12, 25]. Our results confirmed this theory as the level of Cu in urine was significantly increased in our patients compared to control group. This finding was reported by previous investigators [6]. The previous information supports our assumption that the increase in Cu and decrease of Zn may have a role in the pathogenesis of CaOx stones.

In this study, there was a significant increase in the Cu/Zn ratio in urine and plasma samples among CaOx stone patient compared to the control. The increase in Cu/Zn ratio in urine and plasma may contribute to the formation of CaOx stones. According to Khan et al. [26], Cu and Zn can deposit during the formation of stones between the surfaces of crystals of varying composition, resulting in laminations and more brittle lines in stones.

Numerous studies have shown that increasing the reactive oxygen species (ROS) and decreasing the antioxidants cause oxidative stress and contribute to the formation of kidney stones [27–29]. ROS are usually thought to be cytotoxic and can damage lipids, proteins, and DNA [30]. The present work showed that antioxidant markers (GSH and CAT) levels were significantly decreased in CaOx patients compared to control group, while ROS markers including MDA and NO were decreased. These results are confirmed by other investigators [21, 31, 32].

The current study revealed that the gene expression of ERK, P38 and JNK were significantly higher in CaOx patients compared with the control group; these results agree with other researchers [33]. Our results confirmed that MAPK signaling pathway has an essential role in the regulation of CaOx crystallization through, the increase of ROS generation and activation of the JNK, MAPK, and ERK signaling molecules [33].

In our study, the expression of p38, ERK, and JNK showed no correlation with Zn. Regarding the correlation of genes with Cu, ERK and JNK had positive correlation while P38 had negative correlation These observations are in agreement with other investigators [34]. The existence of these correlations in the study group and their absence in control group consolidate our assumption that the disturbance of these biochemical and molecular markers may have a role in the pathogenesis is in CaOx stones.

The present study is not free of limitations; one of these is the small sample size. So, studies with larger sample size are highly recommended to highlight the role of Cu and Zn and their correlations with MAPK signaling pathway in CaOx stones, which may help in understanding the pathogenesis of CaOx stones.

## Conclusion

Changes in the level of Cu and Zn and the disturbance of each of antioxidants markers (CAT and GSH), ROS markers (MDA and NO), and MAPK pathway genes (ERK, P38, and JNK) may have an impact on the pathogenesis of CaOx.

## Data Availability

All data and materials are available if requested.
